# Ectomesenchymal chondromyxoid tumor: Review of literature and a report of a rare case

**DOI:** 10.4103/0973-029X.80021

**Published:** 2011

**Authors:** Mohanty Leeky, TV Narayan, Sadhana Shenoy, Saleha Jamadar

**Affiliations:** *Department of Oral and Maxillofacial Pathology, The Oxford Dental College, Hospital and Research Centre, Bommanahalli, Hosur Road, Bangalore, Karnataka, India*

**Keywords:** Benign, chondromyxoid, ectomesenchymal, tongue, tumor

## Abstract

Ectomesenchymal chondromyxoid tumor (ECMT) is a rare benign intraoral tumor. Clinically, it presents as a slow growing, painless, firm, submucosal sweling exclusively occurring on the anterior dorsum of the tongue. Till date only 40 cases have been reported in literature. Histopathologically the tumor is characterized by a well circumscribed, lobular proliferation of round, polygonal, ovoid or fusiform cells in a net-like pattern in a myxoid to chondromyxoid background. Here, we present a rare case of ECMT occurring in a 7-year-old boy and throw some light on this distinct entity.

## INTRODUCTION

A wide array of tumor or tumor-like lesions affects the tongue, from a simple pyogenic granuloma, granular cell tumor, lipoma to a more complex squamous cell carcinoma. Recently, a new entity, Ectomesenchymal chondromyxoid tumor (ECMT) has been added to the group of lesions affecting the tongue. The uniqueness of this lesion lies in the fact that it mostly affects the anterior dorsum of the tongue.[1]

ECMT is a rare benign tumor occurring as a submucosal swelling on the anterior two third of the dorsum of the tongue. It was first described by Smith *et al*. in 1995 and only 40 cases have been reported till date [Table 1].[1–15]

**Table 1:** Characteristic profile of 41 cases of ECMT reported till date

Author	Year	No. of cases	Age/gender	Site	Peculiar histopathological features	Immunohistochemical profile
Smith *et al*,[1]	1995	19	9 women, 10 men, aged 9-78years	Anterior dorsum tongue	Cytological atypia in the form of nuclear pleomorphism, hyperchromatism and multinucleation. Pseudoinclusion or binucleated cells, pseudocystic spaces with papillary growth pattern, swirling pattern and stromal hyalinization	Strongly positive for GFAP; variably reactive to CD-57; nonreactive to strongly reactive to AE1/AE3; occasionally positive for S-100 and negative for EMA and desmin.
Kannan *et al*,[2]	1996	3	21/M,33/M,51/M	Dorsal tongue, anterior dorsal tongue, anterior dorsal tongue	Few cells showed nuclear pleomorphism, hyperchromatism, multinucleation	Positive for GFAP, S-100, vimentin and CD-57; negative forAEl/AE3 and EMA.
van der Wal and van der Waal[3]	1996	1	25/F	Anterior dorsum tongue	Myxoid lesion focally infiltrating the surrounding muscle tissue	Positive for GFAP, S-100 and vimentin; negative for cytokeratins, SMA, MSA, desmin, CD-57, EMA, and Ulex and factor VIII.
Carlos *et al*,[4]	1999	1	16/M	Left dorsal posterior tongue	–	Positive for GFAP and S-100.
de Visscher *et al*,[5]	2003	2	39/M, 42/F	Anterior dorsal tongue, anterior dorsal tongue	Inclusion of muscle fibers at the periphery of the lesion, atypical cells with nuclear inclusion.	Strongly positive for GFAP, vimentin and S-100; variably positive CD56; focally positive for desmin; negative for pan-keratin cocktail, keratin 8 and 18, SMA and CEA.
Ide *et al*,[6]	2003	1	52/F	Anterior tongue (tip)	Multiple mucin-pooled pseudocysts were filled with round hyalinized globules	Intense positivity for vimentin and GFAP; focally positive for S-lOOand CD-57; Negative for AE1/AE3 and SMA.
Kaplan *et al,*[7]	2004	2	26/M, 57/F	Midline anterior dorsal tongue, left anterior dorsal tongue	Intranuclear psuedoinclusions,	Positive for GFAP and S-100; Negative for SMA, desmin, CD-57, EMA and AE1/3
Woo *et al*,[8]	2005	1	22/F	Midline dorsal tongue	Scant minor salivary gland elements	Negative for GFAP, SMA, desmin, and EMA; strong positivity for S-100 and vimentin; focal positivity for AE1/3, faint positivity for CD-57 and p63.
Goveas *et al*,[9]	2006	1	57/F	Right anterior tongue	Bland spindle and round cells in a myxoid stroma	Strongly positive for GFAP, AEl/3andEMA; focally positive for S-100, vimentin and CK 4; negative for SMA, desmin, CK7, CK20, Calponin, p63, SMMHC and MAC387
Nigam *et al*,[10]	2006	1	30/M	Hard palate	Round to polygonal cells in a chondroid background and stellate cells in a myxoid background, and presence of binucleate cells	Immunoprofile not done
Seckin *et al*,[11]	2008	1	56/F	Anterior Dorsal Tongue	Cells within lacunae where seen in areas of cartilaginous differentiation	Few cells stained for GFAP; negative for Anticytokeratin antibody
Pires *et al*,[12]	2009	3	9/M,16/M, 33/M	Dorsum of the tongue	Pleomorphism and several mitotic figures were seen focally	Strongly positive for GFAP, S-100 and vimentin; focally positive for SMA and desmin.
Portnof *et al*,[13]	2009	1	41/M	Anterior tongue	Striated muscle containing infiltrative tumor, Small collections of larger cells containing foamy cytoplasm.	Strongly positivity for GFAP; focal positivity for SI00 protein and SMA; vimentin, p63, CD99, and Bcl-2 positivity; negative for AE1/AE3, CAM5.2, EMA, calponin, CD34, A103, CD57, and CD117.
Angiero F[14]	2010	1	27/F	Dorsum of the tongue	Neoplastic cells set in a myxoid, chondroid or hyalinized background	Positive for GFAP, S-100 and vimentin; negative for CD- 57, SMA, EMA, Desmin and Cytokeratin
Seo SH *et al,*[15]	2010	2	8/M, 65/M	Posterior dorsum, anterior dorsum of the tongue	Rare mitosis, cup shaped cells and multinucleation, entrapment of cells in the muscle	Negative for GFAP, S-100, SMA, desmin, AE1/3, CD34, p63 and factor VII. Diffusely Positive for vimentin in both the cases, CD56 (1/2 cases) and EMA (1/2 cases)
Present case	2010	1	7/M	Anterior dorsum of the tongue	Cells infiltrating the skeletal muscle, vesiulated and multinucleation	Strongly positive for vimentin; focally positive for SMA and negative for GFAP, S-100 and desmin

The purpose of this paper is to review the literature on ECMT and discuss a rare case occurring in a 7-year-old child.

## REVIEW OF LITERATURE

“Ectomesenchymal chondromyxoid tumor” of the anterior tongue was first described by Smith *et al* in 1995 following review of all myxoid, chondromyxoid, and myoepithelial tongue lesions from the files of the Armed Forces Institute of Pathology in a 24-year period. Of the total cases evaluated, 19 cases did not fulfill the diagnostic criteria for any other intraoral soft tissue chondromyxoid lesion and had similar unique clinicopathological and immunohistochemical features.[1] Following the first report by Smith *et al*, only 21 cases have been reported in the past 15 years taking the total number of cases to 40 in literature till date [Table 1]. The reason for the limited reported cases of ECMT is probably because it being misinterpreted as other similar chondromyxoid lesions like focal mucinosis, soft tissue myxoma, ossifying fibromyxoid tumor, chondroid choristoma, nerve sheath myxoma, myoepithelioma, pleomorphic adenoma and mucocele.

Clinically, ECMT presents as a slow growing asymptomatic swelling exclusively seen on the anterior dorsum of the tongue, however, two cases presenting on the posterior tongue have been documented.[4,15] In addition, a case of ECMT on the hard palate has been reported but due to lack of appropriate documentation to support its diagnosis has been a subject of controversy.[10] The size of the lesion varies from 0.3 to 2.0 cm. Age of affected patients ranges from 9 to 78 years of age, with a mean age of 39 years. Both males and females are affected equally [Table 1].[1,16]

Macroscopic examination of ECMT reveals a submucosal, pale gray to tan to yellow rubbery nodule. Cut surface usually demonstrates a well-circumscribed mass that may have a gelatinous consistency and show foci of hemorrhage. Histopathologically, the lesion is unencapsulated but well circumscribed owing to the compression of the fibrous tissue and the muscle fibers at the periphery of the lesion. The lesional cells are arranged in cords, strands, sheets in a myxoid to chondromyxoid background. Cellular morphology ranges from round, oval, polygonal to spindle shaped. The nuclei are generally rather small and uniform, although some lesions may have foci of nuclear atypia, characterized by variation in nuclear size, evidence of nuclear hyperchromatism, or the presence of binucleated or multinucleated cells and pseudoinclusions.[1,2] Some authors speculate that these areas of suspected atypia could in fact be associated with secondary inflammatory stimuli or aging of the tumors.[2,12] Mitotic figures are rare and necrosis is virtually absent. Few muscle fibers can be focally infiltrated by tumor cells, but does not seem to represent evidence of aggressive behavior.[3,15] Focal areas of inflammation, hemorrhage, few small-caliber blood vessels and partitioning of the tumor lobules by thin bands of fibrous connective tissue can be found.

To aid in the diagnosis of ECMT, Smith *et al*. conducted immunohistochemistry on his 19 cases, which showed strong positivity for GFAP (18 of 19 cases), variably reactive to CD-57/Leu-7 (8 of 9 cases were positive), nonreactive to strongly reactive for cytokeratin (AE1/AE3), positive occasionally for S-100, and negative for Epithelial membrane antigen (EMA) and desmin.[1] Following this many reported cases have used similar immunoprofile for the diagnosis of ECMT with a variable degree of expression of these markers [Table 1].[16]

Various histogeneic concepts have been suggested for the development of ECMT. One such and most accepted hypothesis is it arises directly from neural cells in the tongue or from primitive mesenchymal cells that undergo neural differentiation during tumorigenesis.[1,7,9] Others believe it to be either of myoepithelial or myogenic origin.[8,12] These probable histogenic concepts could be the reason for the variable expression of immunomarkers [Table 1].[1–15]

Treatment of choice of ECMT is conservative surgical excision. But there have been two cases where recurrence has been reported, however were successfully managed in the second surgical intervention.[1] Although all available evidence reinforces the benign nature of ECT, the small number of recurrent cases and the histopathological evidence of foci of pleomorphic hyperchromatic cells, sporadic mitotic figures and muscle infiltration suggest the importance of regular follow-up of patients after treatment.

## CASE REPORT

**Figure 1:**
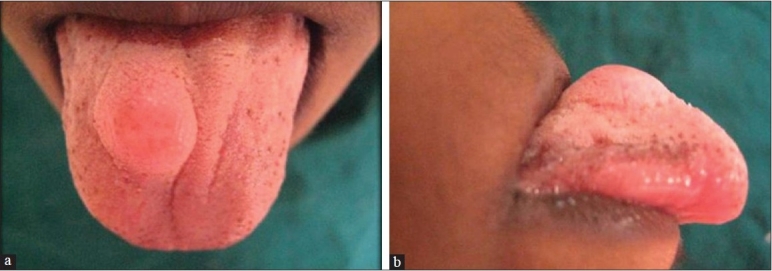
Clinical picture of ectomesenchymal chondromyxoid tumor presenting as a submucosal swelling on the anterior dorsum of the tongue (a, frontal view; b, lateral view)

A 7-year-old boy presented to our dental college with a painless swelling on the anterior dorsum of the tongue of approximately 1 year duration. His medical history was non-contributarory. On oral examination, a solitary well defined nodule measuring 1.5 cm in diameter located on the right side of the dorsum of the anterior two third of the tongue, approximately 1 cm from the tip and 1cm from the lateral border was seen [Figure 1]. There were no secondary changes on the surface of the lesion except for the partial depapillation. On palpation, the nodule was sessile, firm in consistency, mobile and nontender. The reminder of the oral, head and neck examination was unremarkable without discernible lymphadenopathy or neural deficits. Presumptive diagnosis of granular cell tumor/fibroma of tongue were given.

Following the clinical examination, an ultrasound examination was done which revealed a small hyperechoic lesion in the submucosal layer, and a provisional diagnosis of a fibroma was rendered. However, FNAC showed a different picture all together and was highly suggestive of a granular cell tumor. Subsequently, surgical excision was performed following which it was submitted for histopathological examination.

**Figure 2:**
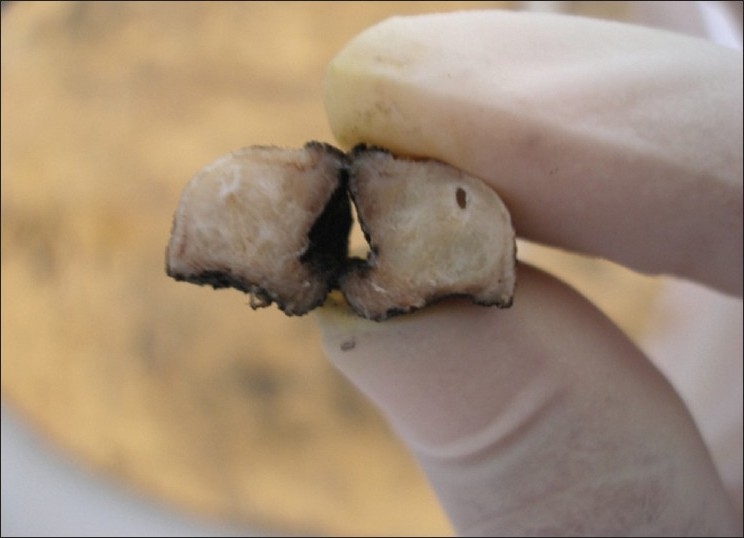
Gross specimen cut surface showing a well-circumscribed soft tissue lesion

**Figure 3:**
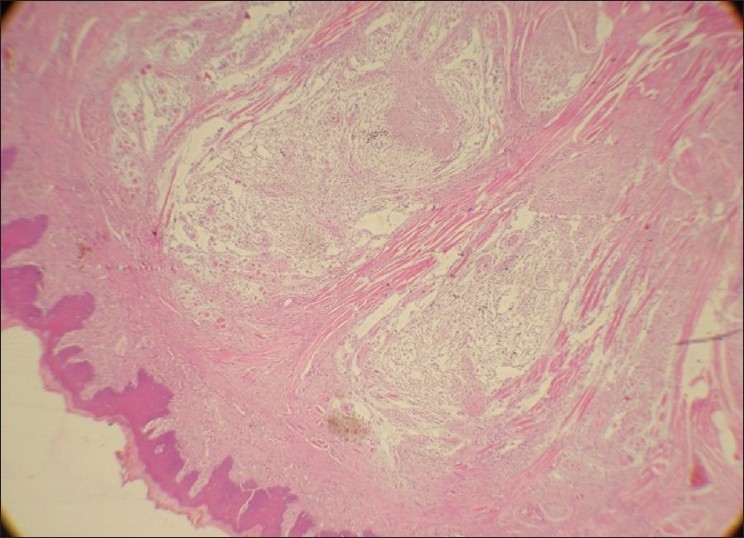
Photomicrograph shows a nonencapsulated but well circumscribed with lobular pattern of arrangement of the tumor (H and E, ×4)

**Figure 4:**
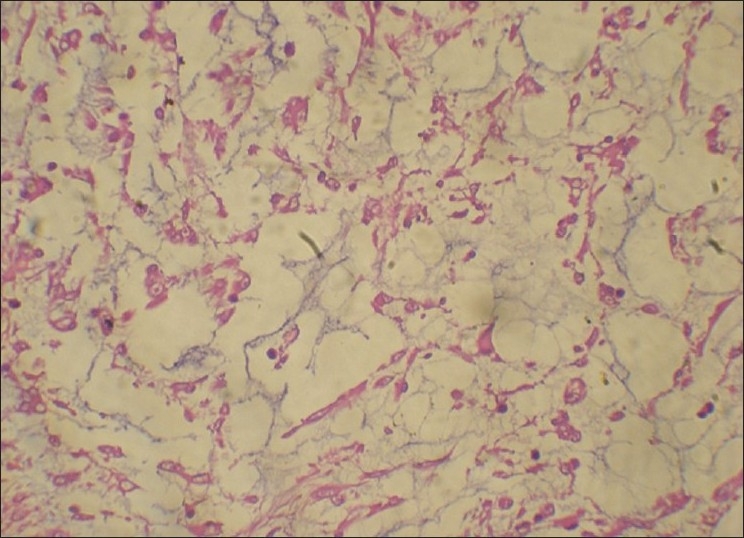
Photomicrograph shows lesional cells arranged in a net-like pattern in a myxoid background (H and E, ×40)

**Figure 5:**
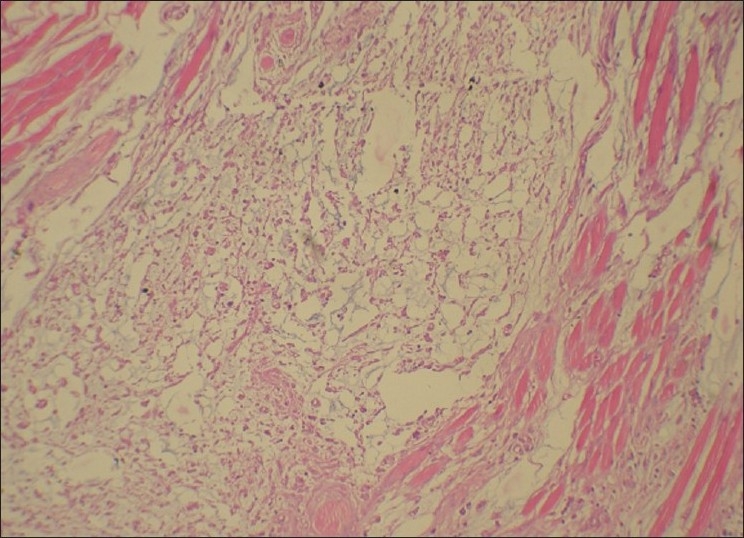
Photomicrograph shows lesional cells infiltrating the striated muscle bundles (H and E, ×20)

On gross examination, the lesion was smooth surfaced, firm in consistency measuring 2.3 × 0.6 × 0.9 cm. The cut surface was white in color and well circumscribed [Figure 2]. Histopathologically, on low-power magnification, the lesion was well circumscribed but unencapsulated showing a lobular growth pattern [Figure 3]. It was separated from the overlying epithelium by a thin layer of loosely compressed connective tissue. On higher magnification, the lesional cells were arranged in the form of cords and strands in a net-like pattern against a myxoid background [Figure 4]. The nuclei were round to oval, showing vesiculation to hyperchromasia. Few multinucleated cells were also seen. However there were no mitosis and no areas of necrosis. The lesional cells were seen infiltrating into the skeletal muscle. No minor salivary glands were found in the lesional tissue. The connective tissue stroma showed few chronic inflammatory cells and thin bands of collagen fibers separating the lesional tissue giving it a lobular configuration. Immunohistochemistry (IHC) was performed and the tumor cells were found to be negative for monoclonal glial fibrillary acidic protein (GFAP), S-100 and desmin. However, the lesional tissue was strongly positive for Vimetin [Figure 5] and focally positive for smooth muscle actin (SMA).

## DISCUSSION

Ectomesenchymal chondromyxoid tumor (ECMT) of the tongue is a relatively rare benign neoplasm that was initially described by Smith *et al*.[1] Clinically, ECMT presents as a slowly growing, painless, firm, well circumscribed submucosal swelling and size of the lesion varies from 0.3 to 2 cm. Its occurrence has been noted in a wide age range from 9 to 78 years; however, most cases have been reported in the adults with no gender predilection [Table 1].[1,16] Histopathologically, the lesion is characterized by well demarcated, lobular proliferation of cells arranged in sheets or cords within a myxoid or chondromyxoid background. These cells are round, polygonal, ovoid, to spindle in shape having round, ovoid or fusiform nuclei; there can be scattered multilobulated or atypical nuclei and cells with nuclear inclusions.[1,2]

The uniqueness of our case lies in the fact that it presented in a 7-year-old boy which is rare a finding as most of the cases of ECMTs reported till date are in adults, with only 5/40 cases reported in the first two decades of life.[1,4,12,15]

Microscopic features of this case paralleled to the findings of other reported cases of ECMTs. The presence of myxoid areas always raises the suspicion of other myxoid lesions such as myoepithelioma, oral focal mucinosis, soft tissue myxoma, glial choristoma, myxolipoma and nerve sheath myxoma which need to be considered in the differential diagnosis.[1]

However these lesions were excluded on the basis of their characteristic histopathological features.

Another finding in our case was that the lesional cells showed an infiltrative pattern into the skeletal muscle component in the periphery of the lesion [Figure 5]. Few researchers have linked this with aggressive behavior; however ECMTs have been found to be indolent.[3,12] Yet complete surgical removal with sufficient depth should be ensured to minimize the chances of recurrence.

**Figure 6:**
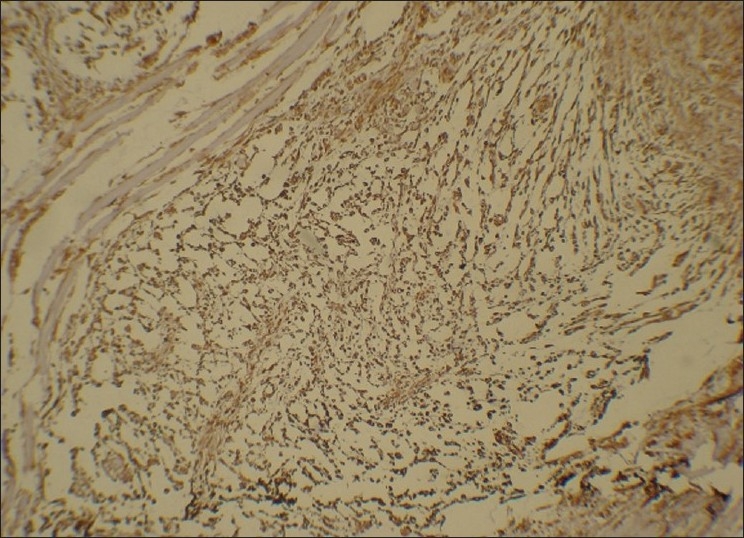
Photomicrograph demonstrates strong vimentin positivity by the lesional cells (× 20)

Although the features in light microscopy are highly suggestive of ECMT, immunohistochemistry has been a helpful adjuvant tool to arrive at the diagnosis of ECMT. Immunohistochemical markers like, polyclonal GFAP, S-100, vimentin have shown highly predictable pattern of positivity for lesional cells. Other markers like cytokeratins, smooth muscle actin (SMA), desmin have shown variable degrees of staining. However, markers of myoepithelial diffentiation such as calponin, smooth muscle myosin heavy chain, p63 do not exhibit immunoreactivity within lesional cells [Table 1].[16] Our case, however, did not express either GFAP or S-100. As in our case, use of monoclonal GFAP has been found to be negative in few previously reported cases and S-100 has been known for its variable staining.[8,11,15] However, the lesional cells showed strong immunoreactivity to vimentin [Figure 6], focal SMA positivity and negative for desmin. These findings of immunoreactivity are consistent with the other studies [Table 1].

This diverse pattern of IHC staining has also cast a shadow on the histogenesis of ECMT which still remains speculative. Most authors agree ECMT is derived from uncommitted ectomesenchymal cells that have migrated from the neural crest although myoepithelial cell and muscle cell origin have been also considered.[1,8,13]

Treatment of choice for ECMT is conservative surgical excision. Only 2 instances of recurrence have been reported, suggesting approximately 7% recurrence rate.[1,16] Our patient was also treated by surgical excision and has been followed up for 5 months with no evidence of recurrence.

## CONCLUSION

This case adds to the limited literature on ECMT and emphasizes the need for this rarely occurring tumor to be considered in the differential diagnosis of nodular lesions affecting the tongue. The occurrence of this lesion in children is even rarer. In contrast to the earlier reported cases, our case showed negative results for GFAP and S-100.The immunoprofile of these lesions need further definition as more cases are added to the literature. Therefore, increased awareness of this distinct entity may lead to a better insight into the clinical behavior and also in understanding its histogenesis.
